# Uncovering deep-rooted conflicts: the role of psychoanalytic psychotherapy in treating athletes' social media-related psychological distress

**DOI:** 10.3389/fspor.2024.1476617

**Published:** 2024-12-11

**Authors:** Paweł Adam Piepiora, Ligiana Mihaela Petre, Jolita Vveinhardt

**Affiliations:** ^1^ Faculty of Physical Education and Sports, Wroclaw University of Health and Sport Sciences, Wrocław, Poland; ^2^ Faculty of Psychology and Educational Sciences, University of Bucharest, Bucharest, Romania; ^3^ Institute of Sport Science and Innovations, Lithuanian Sports University, Kaunas, Lithuania

**Keywords:** athletes, hate speech, mental health, psychoanalytic psychotherapy, social media, sports psychology, wellbeing

## Abstract

The use of social media by athletes can support them in difficult moments, but it can also become a source of negative emotions and psychological distress. This perspective critically examines psychoanalytic psychotherapy as a method for restoring athletes' psychological well-being after experiencing negative effects from social media use. The paper characterizes the key elements of psychoanalytic psychotherapy relevant to athletes, discusses the role of the psychoanalytic psychotherapist in working with athletes and describes the specifics of the psychoanalytic therapeutic process in this context. The potential advantages of psychoanalytic approaches over cognitive-behavioral methods are examined in treating the psychological impacts of problematic social media use among athletes. The analysis suggests that hate speech and negative online interactions can activate athletes' unprocessed life experiences rooted in early developmental stages. Psychoanalytic psychotherapy may offer a particularly effective method for restoring athletes' psychological wellbeing in such cases by addressing deep-rooted intrapsychic conflicts. However, a comprehensive approach integrating multiple therapeutic modalities is recommended to address the complex challenges athletes face in the digital age. This perspective acknowledges limitations in current research and suggest directions for future studies to develop and validate tailored interventions for athletes grappling with social media-related psychological distress.

## Introduction

Social media has become an indispensable communication tool for athletes, significantly amplifying their social reach and influence (1, 2). This digital presence not only bolsters athletes' popularity in pop culture but also facilitates the promotion of their own brand and products. Social media enables real-time fan engagement. The constant stream of content from training sessions, competitions, and personal moments fosters a sense of intimacy between athletes and their followers (3). This unprecedented access creates an ideal environment for targeted advertising campaigns. By consistently sharing valuable content, athletes cultivate fan loyalty and encourage active interaction. Therefore, social media has emerged as a cornerstone of sports marketing, offering opportunities for market research and brand development (4).

While social media offers numerous benefits, it also presents significant challenges for athletes, including potential reputation damage, constant scrutiny, and psychological distress from negative interactions (5–7).

The psychological challenges athletes face in the digital era necessitate professional mental health intervention (8). While sports psychology interventions generally yield positive effects, the pervasive and complex nature of social media-related issues may require more intensive therapeutic modalities. For many athletes, cognitive-behavioral therapy (CBT) can effectively address specific psychosocial issues related to social media use (9). However, some athletes present with more profound psychopathology that may be exacerbated by social media exposure (10).

This article aims to provide a critical perspective on the potential of psychoanalytic psychotherapy as an intervention for restoring athletes' psychological wellbeing in response to the adverse effects of social media engagement. Despite its long-standing history and evolving applications, psychoanalytic psychotherapy remains an undervalued approach in addressing the complex psychological challenges faced by athletes in the 21st century. Therefore, this paper posits that there is a legitimate need to disseminate knowledge about psychoanalytic psychotherapy within the field of sport psychology, particularly in the context of digital-age stressors. By exploring the unique contributions of psychoanalytic theory and practice to athlete mental health, this viewpoint seeks to broaden the discourse on effective therapeutic interventions in sports psychology.

## Characteristics of psychoanalytic psychotherapy

Psychoanalytic psychotherapy is a prominent approach in therapeutic work with the patient-athlete. This method is predicated on the concept of intrapsychic conflict among the id (instinctual drives), superego (internalized social and cultural norms), and ego (the mediating aspect of personality), as originally posited Freud (11). The primary objective of psychoanalytic psychotherapy for athletes is to develop an integrated personality structure, wherein the id, ego, and superego, function harmoniously (12). While psychoanalytic psychotherapy can address more severe psychopathology, it maintains a non-directive stance. This approach eschews prescriptive tasks, injunctions or explicit advice-giving to the athlete-patient (13).

The approach emphasizes the analysis of the athlete's dreams, fantasies, memories, and free associations. It prioritizes uncovering the unconscious etiology of presenting problems rather than solely addressing symptomatic manifestation. In the context of athletes grappling with social media-related stressors, psychoanalytic psychotherapy offers a unique lens through which to examine the deeper, often unconscious, impact of digital interactions on the psyche (14).

Psychoanalytic psychotherapy becomes an integral component of the athlete's psychological milieu over an extended period, necessitating the development of a robust therapeutic alliance characterized by trust and emotional intimacy (15). The efficacy of psychoanalytic therapy extends beyond symptom reduction to encompass a global enhancement in subjective well-being and interpersonal functioning (16, 17).

The fundamental aim of psychoanalytic psychotherapy is the restructuring of personality organization. Early relational experiences with primary caregivers and formative life events, often serve as the source of intrapsychic conflicts (18). These experiences and their associated fantasies generate unconscious relational patterns that influence self-perception and interpersonal dynamics throughout the lifespan. Through the psychoanalytic process, patients gain insight into these unconscious mechanisms and conflicts, thereby attaining greater agency over their psychological functioning and life trajectories (19, 20).

For athletes, psychoanalytic psychotherapy can be particularly beneficial in addressing deep-seated psychological barriers that may impede optimal functioning. By exploring unconscious conflicts and enhancing self-awareness, athletes can potentially overcome performance anxiety, improve focus, and develop a more integrated sense of self (15).

This approach is particularly indicated for athletes experiencing a wide spectrum of psychosocial distress or functional impairment. These may include social withdraw, relational conflicts, intimacy avoidance, occupational underperformance, unresolved trauma, intrusive recollections, generalized mistrust, social maladjustment, complicated grief, creative inhibition, and academic or professional challenges (21, 22). Additionally, it can address specific psychopathological conditions such as anxiety disorders, depressive symptomatology, obsessive-compulsive spectrum disorders (23, 24), somatoform disorders, and specific phobic disorders, which can significantly impair athletic performance and quality of life (25, 26).

Psychoanalytic psychotherapy offers a comprehensive framework for addressing these multifaceted issues, fostering psychological resilience and adaptive coping mechanisms essential for long-term athletic success and personal well-being (27). It can be particularly valuable in exploring the unconscious dynamics that may underlie performance anxiety, identity conflicts related to athletic roles, and the psychological impact of injuries or career transitions (15).

## The role of the psychoanalytic psychotherapist

Psychoanalytic psychotherapy aims to bring relief to the athlete's suffering. However, the psychotherapist does not focus on relieving the symptoms of the suffering, but instead seeks the causes of the suffering in the unconscious, going back to the patient's earliest experiences and analyzing their impact on their current relationships and behavior. The role of the psychotherapist is to elucidate the athlete's inner conflicts and to facilitate their resolution. To this end, the psychotherapist traces their early childhood origins (28). Regression, as a therapeutic mechanism, plays an important role in accessing athlete's unconscious psychic content. This process involves the unconscious projection of significant relational patterns onto the therapeutic relationship. By analyzing these transferential dynamics, the therapist gains insight into the athlete's internalized object relations and relational schemas, providing a window into their interpersonal history and current relational challenges (29). Transference is another crucial aspect of psychoanalytic therapy. In transference, the athlete unconsciously transfers their own emotional reactions and mental states to the therapist. Thus, they treat the therapist as someone close to them. It is used to understand how the patient's interpersonal relationships were in the past (30). Countertransference, the therapist's emotional response, is also significant. These are the feelings experienced by the therapist, which are the result of both their own ways of feeling and the emotional response aroused by the particular patient and the issues presented by the patient. Importantly, the role of the therapist is not to undertake any behavior as a result of their countertransference. Instead, the therapist should experience and observe their countertransference, reflect on it to transform it into a verbal message. This enables the athlete to see new dimensions of their own feelings and behaviors that they may not have been aware of before (31).

## The therapeutic process in psychoanalytic work with athletes

The psychoanalytic therapeutic encounter provides a unique space for athletes to explore their interpersonal difficulties, and process negative emotions. The psychotherapist may ask questions about the athlete's development history, current experiences, and intrapsychic dynamics. In working with the athlete, the psychoanalytic emphasizes the therapist's stance of impartiality, neutrality, and authenticity, which facilitates the transferential process (32). The exchange between psychotherapist and patient differs from quotidian conversation. The psychotherapist refrains from imposing topics, instead allowing the athlete's free associations to guide the session's content. Periods of silence are viewed as therapeutically valuable, providing opportunities for introspection and the emergence of unconscious material. This non-directive approach allows for a fluid therapeutic process, unbounded by rigid patterns. The psychoanalytic psychotherapist employs active listening and judicious inquiry, while maintaining a non-directive stance. The psychoanalytic psychotherapist also observes athlete's non-verbal communication and paralinguistic cues, analyzing the athlete's relational patterns and defense mechanisms (33). The psychotherapist behaves with restraint, does not talk about themselves, and avoid physical contact with the patient. This neutral attitude is meant to foster a “digging inside” of the athlete. It does not refer to who the psychotherapist is, what they think (34). Through this process, the psychotherapist recognizes that the intense feelings the athlete experiences in the therapy are rooted in their past experiences with other people and past situations. Analyzing the transference helps the athlete to understand their feelings, re-build more satisfying relationships with the important people in their life and allows for deeper changes in the personality (35).

The psychotherapist pays attention to the overt content of the athlete's statements and their unconscious meanings. On this basis, and based on what they already know about the patient, their history and the defense mechanisms they use, the psychotherapist formulates interpretations which they present to the patient. The psychotherapist speaks up when they have something important to say (36). Psychotherapists vary in temperament and working style, so that the number of sentences they utter during a session may also vary. The use of psychoanalytic techniques allows the therapist to repeatedly confront the athlete with difficult issues until internal barriers are broken down and the athlete opens to psychological change. This lengthy process offers the chance to re-evaluate emotionally and transform thinking in a manner where the effect proves to be long-lasting (37).

## The duration of psychoanalytic psychotherapy

While the average duration of psychoanalytic psychotherapy is approximately two years, some athletes may engage in treatment for extended periods, ranging from five to seven years or more (38). Maladaptive psychological patterns, often deeply ingrained, require substantial time and therapeutic effort to modify. Consequently, the transformation of longstanding intrapsychic and interpersonal dynamics necessitates a prolonged and intensive therapeutic engagement (39). Contemporary psychoanalytic practice typically recommends a minimum of three to four regular sessions per week, although the precise frequency may be tailored to the specific needs and circumstances of the athlete and the therapist's clinical judgement (40).

Psychoanalytic psychotherapy should ends when the therapist and the athlete are convinced that termination is possible and advisable, but there may be situations that are difficult for the athlete, in which they experience painful feelings. These may prompt them to discontinue psychotherapy. If the athlete makes a sudden decision to discontinue psychotherapy, the psychotherapist will seek to understand the reason for the athlete's decision and interpret it (41).

## Discussion

In psychoanalytic psychotherapy, gaining access to the unconscious is considered crucial for therapeutic progress (42). The psychoanalytic method employs techniques to elucidate unconscious content by analyzing of free associations, dreams, and transference dynamics (43). Psychoanalytic psychotherapy addresses the role of unconscious processes and childhood experiences (44). In the context of athletes experiencing psychological distress because of online hate speech and negative social media, psychoanalytic approaches may be particularly efficacious. The method's capacity to trace current psychological complexes to their developmental origins allows for a comprehensive understanding of how early experiences may predispose athletes to vulnerability in the face of public criticism (15, 45).

The cognitive-behavioral psychotherapy method is effective in the treatment of social media addiction (46). Social media addiction, a subset of addiction disorders, involves increasing time spent on social media, shaping worldviews based on online trends, and difficulties in offline relationships (47, 48). This process often correlates with heightened social anxiety, impaired self-esteem, increased neuroticism and high vulnerability to stress (49). The phenomenon may be more prevalent among athletes who are more oriented towards self-promotion rather than sport performance.

While psychoanalytic and cognitive-behavioral approaches have garnered significant empirical support in athletic contexts, there are other methods of psychotherapy, including systemic, and humanistic—experiential approaches. There is an ongoing systematic search for new methods of psychotherapy referring to various case studies and quasi-experiments (50). However, so far, the results of studies on the effectiveness of other methods of psychotherapy among athletes besides psychoanalytic and cognitive-behavioral remains less extensively documented in the current literature.

Recent developments in neuropsychoanalysis and attachment-based therapies have begun to bridge the gap between psychodynamic approaches and neuroscientific findings, potentially offering new avenues for understanding and treating psychological issues in athletes (51). These integrative approaches may prove particularly valuable in addressing the complex interplay between early developmental experiences, neurobiological factors, and the unique pressures of athletic careers.

## Limitations of perspective

The present analysis is constrained by several notable limitations. The analysis is circumscribed by the authors' expertise in psychoanalytic psychotherapy and specific case studie, potentially overshadowing alternative therapeutic approaches. The reliance on case studies, limits generalizability to broader athletic populations. The perspective may be subject to bias inherent in the psychoanalytic framework, which, which has faced critiques regarding empirical validation (52). The exclusion of quantitative outcome studies and systematic reviews limits definitive conclusions about the comparative efficacy (53). Lastly, the rapid evolution of digital technologies may outpace the development of therapeutic interventions, potentially limiting the applicability of traditional psychoanalytic concepts to contemporary challenges faced by athletes in the digital age.

## Directions for further research

To address limitations and advance the field of sports psychology, we propose several key research directions:

1. Conduct comparative efficacy of various psychotherapeutic approaches for athletes.
2. Explore the integration of neuroscientific findings with psychotherapy for athletes.
3. Develop and validate sport-specific psychotherapeutic protocols
4. Implement longitudinal studies to assess long-term impact on athletic performance and well-being.
5. Investigate cultural adaptations of psychotherapeutic approaches for diverse athletic populations.
6. Explore technology-based interventions and telepsychology for athlete scalable mental health support, particularly in addressing social media-related challenges.
7. Combine quantitative and qualitative methods to capture the nuanced experiences of athletes in psychotherapy.
8. Further investigate mentalization-based interventions and attachment-informed therapies for enhancing athletes' psychological resilience and interpersonal functioning.
9. Foster interdisciplinary collaboration between sports psychologists, clinical psychologists, neuroscientists, and sports medicine professionals.
10. Conduct systematic reviews and meta-analyses to identify knowledge gaps and guide future research priorities.

By pursuing these research directions, the field of sports psychology can develop a more nuanced, evidence-based understanding of effective psychological interventions for athletes, ultimately enhancing both athletic performance and overall well-being.

## Conclusions

The negative consequences of social media in contemporary athletic contexts presents specific psychological challenges, particularly when athletes are exposed to hate speech and negative commentary. These digital interactions can activate or exacerbate athletes' unprocessed psychological conflicts, often rooted in early developmental experiences. In such cases, psychoanalytic psychotherapy emerges as a potentially effective method for restoring and enhancing athletes' psychological well-being.

However, psychoanalytic psychotherapy is not a panacea for all psychological challenges faced by athletes in the digital age. The complexity and diversity of athletes' experiences necessitate a nuanced, multimodal approach to psychological intervention. While psychoanalytic methods may be particularly suited to addressing deep-rooted psychological conflicts activated by social media interactions, other evidence-based approaches, such as cognitive-behavioral therapy, may offer complementary benefits, particularly in addressing more immediate behavioral and cognitive aspects of social media use. A comprehensive approach to athlete mental health should consider the integration of multiple therapeutic modalities. Future research should focus on developing and validating tailored interventions that address the specific challenges posed by social media in athletic contexts, while also considering individual differences in psychological structure and developmental history.

## Data Availability

The data analyzed in this study is subject to the following licenses/restrictions: The original contributions presented in the study are included in the article/Supplementary Material, further inquiries can be directed to the corresponding author. Requests to access these datasets should be directed to jolita.vveinhardt@lsu.lt
